# Paclitaxel drug-coated balloon-only angioplasty for de novo coronary artery disease in elective clinical practice

**DOI:** 10.1007/s00392-022-02106-y

**Published:** 2022-09-14

**Authors:** Ioannis Merinopoulos, Tharusha Gunawardena, Natasha Corballis, U Bhalraam, Tim Gilbert, Clint Maart, Paul Richardson, Alisdair Ryding, Toomas Sarev, Chris Sawh, Sreekumar Sulfi, Upul Wickramarachchi, Trevor Wistow, Mohamed O. Mohamed, Mamas A. Mamas, Vassilios S. Vassiliou, Simon C. Eccleshall

**Affiliations:** 1 Department of Cardiology, Norfolk and Norwich University Hospital, Norwich, UK; 2 Norwich Medical School, University of East Anglia, 2.06 Bob Champion Research and Education Building, Norwich, NR4 7TJ UK; 3 Division of Molecular and Clinical Medicine, University of Dundee, Dundee, UK; 4 University of Keele, Keele, Staffordshire UK

**Keywords:** Drug-coated balloon, De novo disease, Stable angina

## Abstract

**Objective:**

We aimed to investigate the safety of drug-coated balloon (DCB)-only angioplasty compared to drug-eluting stent (DES), as part of routine clinical practice.

**Background:**

The recent BASKETSMALL2 trial demonstrated the safety and efficacy of DCB angioplasty for de novo small vessel disease. Registry data have also demonstrated that DCB angioplasty is safe; however, most of these studies are limited due to long recruitment time and a small number of patients with DCB compared to DES. Therefore, it is unclear if DCB-only strategy is safe to incorporate in routine elective clinical practice.

**Methods:**

We compared all-cause mortality and major cardiovascular endpoints (MACE), including unplanned target lesion revascularisation (TLR) of all patients treated with DCB or DES for first presentation of stable angina due to de novo coronary artery disease between 1st January 2015 and 15th November 2019. Data were analysed with Cox regression models and cumulative hazard plots.

**Results:**

We present 1237 patients; 544 treated with DCB and 693 treated with DES for de novo, mainly large-vessel coronary artery disease. On multivariable Cox regression analysis, only age and frailty remained significant adverse predictors of all-cause mortality. Univariable, cumulative hazard plots showed no difference between DCB and DES for either all-cause mortality or any of the major cardiovascular endpoints, including unplanned TLR. The results remained unchanged following propensity score-matched analysis.

**Conclusion:**

DCB-only angioplasty, for stable angina and predominantly large vessels, is safe compared to DES as part of routine clinical practice, in terms of all-cause mortality and MACE, including unplanned TLR.

**Graphic abstract:**

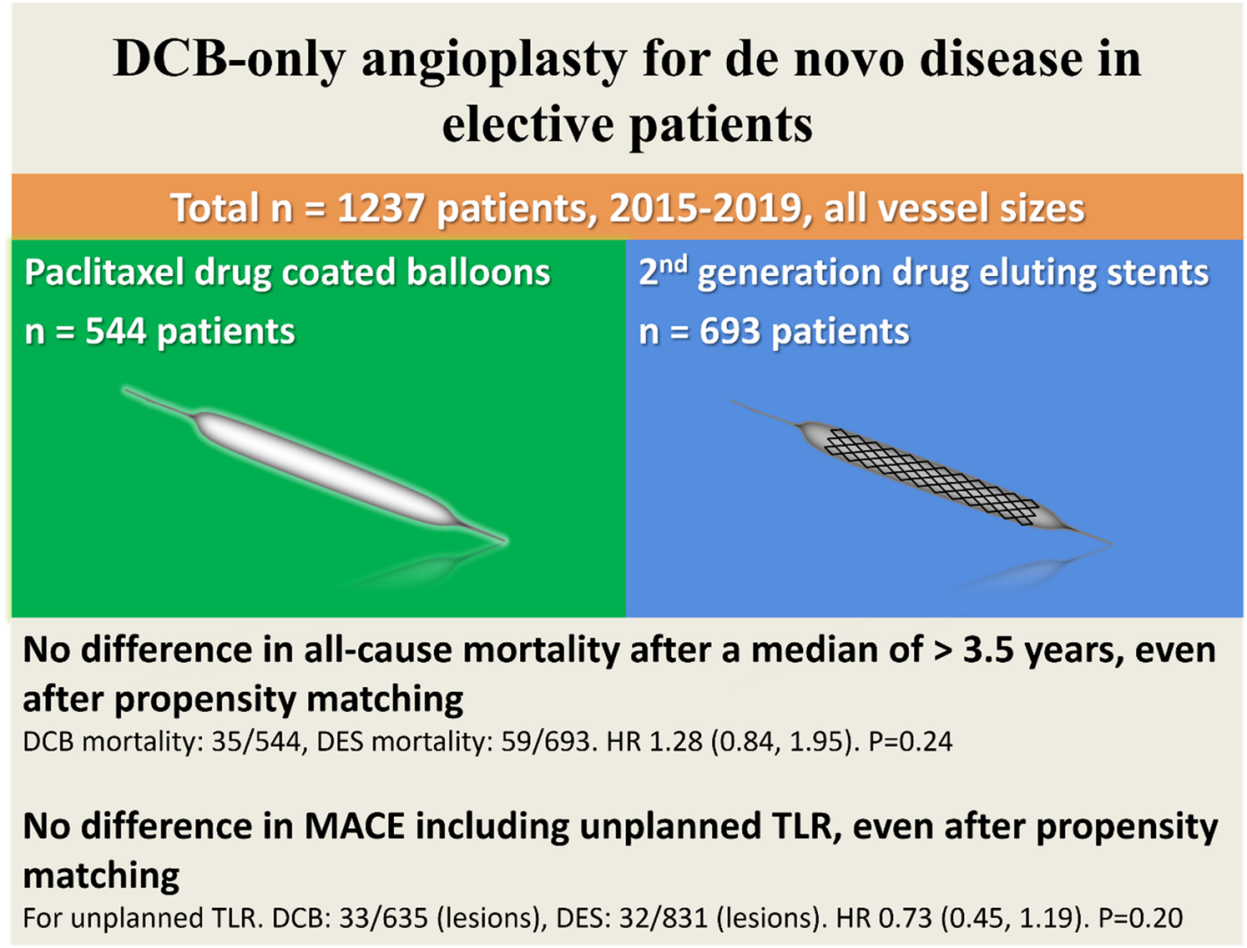

## Introduction

Implantation of second-generation drug-eluting stents (DES) is the current guideline-recommended treatment strategy for de novo coronary artery disease [1]. Stents were initially developed to treat the limitations of plain old balloon angioplasty related to flow-limiting dissections and acute vessel recoil [2]. However, the persistence of stent-related complications, such as stent thrombosis and in-stent restenosis, stimulated the concept of ‘leaving nothing behind’ [2]. Drug-coated balloons (DCB) were developed to combine the benefits of local drug treatment without the complications of stent implantation in cases where stenting was not mandated after initial angioplasty [3]. Currently, DCBs represent an alternative, emerging treatment strategy with supportive evidence in specific groups, such as patients with in-stent restenosis, high-bleeding risk or small vessel disease [4, 5]. Randomised data have demonstrated maintained safety and efficacy of DCB vs DES for de novo small vessel coronary artery disease [6–8]. However, there are no data about the safety of DCB-only angioplasty as part of routine clinical practice and there are limited data about the safety of DCB in de novo large vessels [9]. There are no data evaluating if it is possible and safe for DCB-only angioplasty to become part of a routine PCI treatment strategy.

Previous work from our group (SPARTAN DCB) demonstrated that there is no evidence of increased late mortality associated with paclitaxel DCB, and indeed better survival with DCB in the propensity score-matched cohort [10]. However, that analysis excluded patients with previous percutaneous coronary intervention (PCI) and patients with different PCI strategy in subsequent procedures compared to index (i.e. patients treated with DES initially and then later treated with DCB or vice versa were excluded). Even though that study design was necessary in order to achieve group homogeneity and investigate a true potential effect of paclitaxel, it poses a limitation in terms of generalisability. In the current study we have addressed this limitation by including patients with previous PCI and subsequent PCI irrespective of initial PCI strategy.

In this study, we aimed to explore the safety of DCB-only angioplasty judged by overall mortality, as well as major cardiovascular endpoints, in routine clinical practice for stable, de novo coronary artery disease in all vessel sizes.

## Methods

**Fig. 1:**
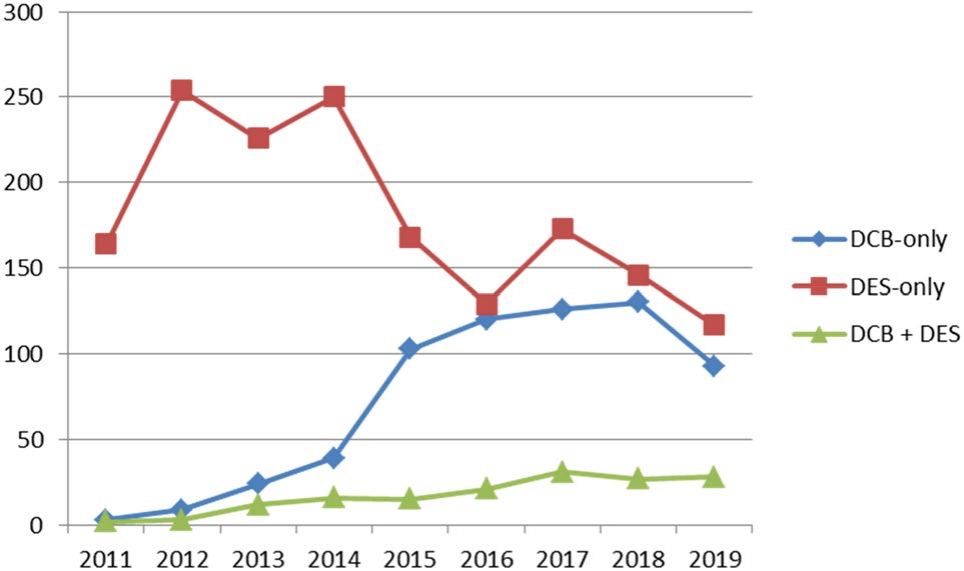
Yearly usage of DCB and DES in patients with first presentation with stable angina and de novo disease

The paclitaxel drug-coated balloon-only angioplasty for stable de novo coronary artery disease in routine clinical practice study was an investigator-initiated, single-centre, cohort study. In our institution, patients undergoing PCI are prospectively entered in a dedicated clinical database. Following approval from the Northwest Haydock (17/NW/0278), UK research ethics committee and Institutional Board approval by the Norfolk and Norwich University Hospital, we retrospectively surveyed our clinical database to identify all patients whose first entry was for stable, de novo coronary artery disease, up to November 2019. The confidentiality advisory group waived the need for patient consent due to the retrospective nature of our study (17/CAG/0145). In our institution, the use of DCB has steadily increased with a complementary decrease in second-generation DES use over the last ten years. From 2015 onwards more than 100 patients per year (more than about 40% of patients), with first presentation of stable angina and de novo disease, were treated with DCB-only angioplasty (Fig. 1). We included patients from January 2015 to November 2019 to allow a similar number of patients to be included from each group, without affecting the follow-up period in each group. Clinical and angiographic data were obtained from our prospectively collated database supplemented with data from electronic hospital records as required. All angiograms were reviewed by an expert operator to confirm accuracy of treatment strategy, classify bifurcation disease, coronary dissection post-DCB implantation, and determine target lesion revascularisation. A lesion was defined as a bifurcation if there was a side branch more than 2 mm in diameter within 5 mm of the lesion. MEDINA subtypes 1.1.1, 1.0.1 and 0.1.1 were considered as true bifurcations [11]. The vessel diameter was considered as the largest pre-/post-dilatation balloon, DCB or DES used and lesion length was based on the DCB or DES length.

The primary endpoint was all-cause mortality. The secondary endpoints were cardiovascular mortality, acute coronary syndrome (ACS), stroke or transient ischaemic attack, major bleeding and target lesion revascularisation. Patient outcomes were obtained from the Hospital Episode Statistics from NHS digital. Hospital Episode Statistics is a data warehouse containing details of all admission, outpatient appointments and accident and emergency attendances at NHS hospitals in England. Supplementary Table 1 demonstrates the ICD-10 diagnostic codes used to identify patients’ outcomes. All deaths were classified as cardiovascular or non-cardiovascular by an adjudication committee according to academic research consortium two consensus [12]. We used the validated Hospital Frailty Risk Score based on ICD-10 diagnostic codes to calculate the patients’ frailty index [13].

Statistical analysis was undertaken in R (version 4.2). Nominal variables are reported as counts (percentages) and compared by the Chi-square test. Variables that were not normally distributed, as assessed by the Kolmogorov and Shapiro tests, are reported as median (interquartile range (IQR)) and compared with non-parametric tests (Wilcoxon rank sum test). Univariable Cox regression analyses were performed to identify predictors of mortality and cardiovascular mortality. Predictors with *p* value < 0.05 were introduced into the multivariable Cox regression model. Data are reported as hazard rations (HRs) with 95% confidence intervals. A *p* value < 0.05 was considered significant. Cumulative hazard plots were used to compare patient outcomes. Comparisons were performed by the log-rank test.

## Results

**Fig. 2:**
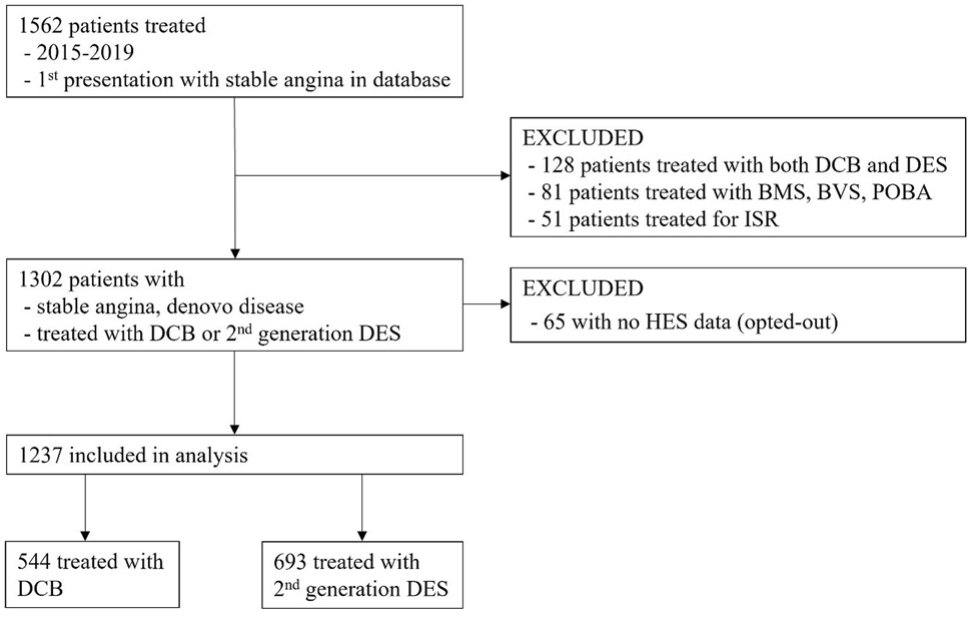
Consort diagram indicating how the final population included in this study was identified

A total of 544 consecutive patients (640 de novo lesions) treated with paclitaxel DCB and 693 consecutive patients (831 de novo lesions) treated with 2nd-generation DES were identified (Fig. 2). The median age was 69 (IQR 61–75) for both groups. Male patients accounted for 79% of the DCB and 78% of the DES group. The groups were well balanced in baseline patient characteristics as shown in Table 1. The only difference was that the DES group had significantly more patients with chronic obstructive pulmonary disease.

**Table 1:** Baseline patient characteristics of patients treated with DCB or DES

Characteristic	DCB, *N* = 544	DES, *N* = 693	*p* value
Age, median (IQR)	69 (61–75)	69 (61–75)	0.61^1^
Male, *n* (%)	429 (79)	541 (78)	0.74^2^
Current/ex-smoker, *n* (%)	336 (62)	455 (66)	0.11^2^
Hypercholesterolaemia, *n* (%)	186 (34)	224 (32)	0.49^2^
Hypertension, *n* (%)	307 (56)	397 (57)	0.76^2^
Peripheral vascular disease, *n* (%)	24 (4.4)	33 (4.8)	0.77^2^
Cerebrovascular disease, *n* (%)	42 (7.7)	37 (5.3)	0.089^2^
Myocardial infarction, *n* (%)	93 (17)	123 (18)	0.76^2^
Percutaneous coronary intervention, *n* (%)	79 (15)	86 (12)	0.28^2^
Coronary artery bypass graft, *n* (%)	47 (8.6)	56 (8.1)	0.72^2^
Atrial fibrillation, *n* (%)	56 (10)	52 (7.5)	0.084^2^
Heart failure, *n* (%)	18 (3.3)	20 (2.9)	0.67^2^
Chronic obstructive pulmonary disease, *n* (%)	18 (3.3)	44 (6.3)	**0.015**^2^
Diabetes, *n* (%)	125 (23)	153 (22)	0.71^2^
Family history, *n* (%)	148 (27)	174 (25)	0.40^2^
eGFR (ml/min/1.73 m^2^) median (IQR)	79 (66–91)	78 (67–91)	0.85^1^
Frailty, *n* (%)			> 0.99^3^
Low	541 (99)	688 (99)	
Intermediate	3 (0.6)	5 (0.7)	
High	0 (0)	0 (0)	

Data are *n* (%) or median (IQR)

*DCB* drug-coated balloon, *DES* drug-eluting stent, *eGFR* estimated glomerular filtration rate, *eGFR* estimated glomerular filtration rate

^1^Wilcoxon rank sum test

^2^Pearson's Chi-squared test

^3^Fisher's exact test

^4^Wilcoxon rank sum exact test

Bold indicate significant result

The angiographic characteristics of the target vessels treated are shown in Table 2. The groups were well balanced in terms of prognostically significant vessels targeted (LMS or LAD and multivessel PCI). The DES group had more patients with large vessel treated, while the DCB group had more patients with true bifurcations. The DES group had significantly more patients on dual antiplatelet therapy (DAPT), while the duration of DAPT was significantly longer in the DES group as well.

**Table 2:** Angiographic characteristics of target vessels treated with DCB or DES

Characteristic	DCB, *N* = 544	DES, *N* = 693	*p* value
*Vessels treated, n (%)*			0.006^2^
LMS	15 (2.8)	27 (3.9)	
LAD	309 (57)	376 (54)	
LCx	104 (19)	98 (14)	
RCA	111 (20)	172 (25)	
Graft	5 (0.9)	20 (2.9)	
Multivessel PCI, *n* (%)	51 (9.4)	83 (12)	0.14^2^
Patients with true bifurcation disease, *n* (%)	63 (12)	56 (8.1)	**0.038**^2^
Patients with vessel treated ≥ 3 mm	398 (73)	594 (86)	** < 0.001**^2^
Dual antiplatelet therapy	518 (95.2%)	681 (98.3%)	** < 0.002**^2^
Duration of dual antiplatelet therapy, Median (IQR) days	30 (29, 31)	365 (364, 365)	** < 0.001**^1^
*Lesions*			
De novo lesions treated	DCB (640)	DES (831)	
True bifurcation, *n* (%)	64 (10)	55 (6.6)	**0.02**^2^
Vessel diameter (mm), Median (IQR)	3.00 (2.75–3.50)	3.50 (3.00–3.75)	** < 0.001**^1^
Lesion length (mm), median (IQR)	20 (20–30)	24 (18–38)	**0.043**^1^
*Dissection grade post-DCB* [14]			
A	20 (3.1%)	n/a	
B	278 (43.4%)	n/a	
C	5 (0.8%)	n/a	
D	3 (0.5%)	n/a	

*DCB* drug-coated balloon, *DES* drug-eluting stent, *LMS* left main stem, *LAD* left anterior descending, *LCx* left circumflex, *RCA* right coronary artery, *TIMI* thrombolysis in myocardial infarction * indicates significant result

^1^Wilcoxon rank sum test

^2^Pearson's Chi-squared test

^3^Fisher's exact test

^4^Wilcoxon rank sum exact test

Bold indicate significant result

**Fig. 3:**
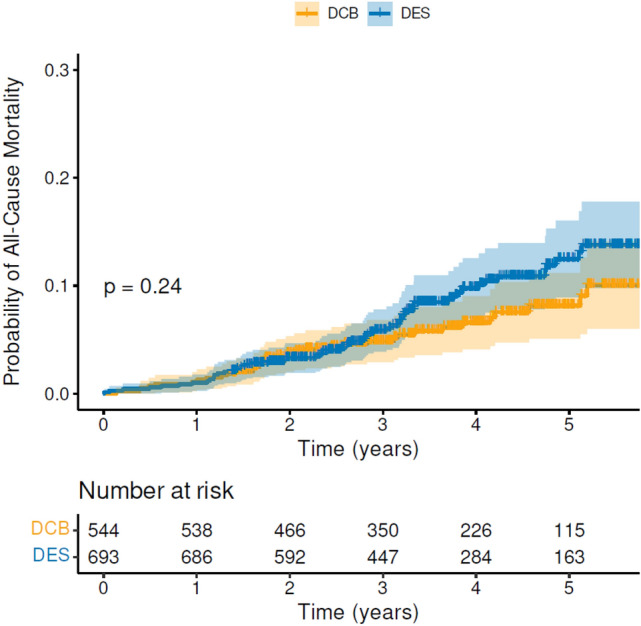
Cumulative hazard plot of all-cause mortality for DCB versus 2nd-generation DES with numbers at risk shown below the graph. *DCB* drug-coated balloon, *DES* drug-eluting stent

The median follow-up of patients in the DCB group was 3.7 years (IQR 2.5–4.8), while the median follow-up in the DES group was 3.6 years (IQR 2.6–4.9). There was no evidence of increased all-cause mortality (Fig. 3) associated with paclitaxel DCB for de novo coronary artery disease compared to 2nd-generation DES. The mortality rate was 35/544 in the DCB group versus 59/693 in the DES group (HR = 1.28; CI 0.84–1.95; *p* = 0.24). Furthermore, there was no difference in any of the secondary endpoints, cardiovascular mortality, ACS, stroke/TIA, major bleeding or unplanned TLR (Supplementary Fig. 1). Univariable Cox regression analysis identified the following adverse prognostic factors for all-cause mortality: increasing age, coronary artery bypass (CABG), heart failure, atrial fibrillation (AF), diabetes, decreasing estimated glomerular filtration rate (eGFR) and frailty. Hypercholesterolaemia was associated with better survival (Table 3). On multivariable Cox regression analysis only age and frailty remained significant predictors of mortality (Table 4). Finally, in terms of short-term safety, one patient in the DCB group had acute vessel closure a few hours later and needed to return urgently to the lab. Two patients in the DES group returned urgently to the lab within 72 h (one with subacute stent thrombosis and one with stent edge disruption requiring further stent). No other patient returned urgently to the lab within seven days in either group.

**Table 3:** Results of univariable Cox regression analysis for all-cause mortality

Mortality (Univariate)	*N*	Forest Plot	HR (95% CI)	*p* value
DCB/DES [DES]	1237		1.28 (0.84 to 1.95)	0.24
Age	1237		1.10 (1.07 to 1.12)	** < 0.001**
Gender [female]	1237		1.56 (1.00 to 2.45)	0.050
Smoking status [current/ex-smoker]	1230		1.26 (0.81 to 1.95)	0.31
Hypercholesterolaemia	1237		0.44 (0.26 to 0.74)	**0.002**
Hypertension	1237		1.42 (0.93 to 2.17)	0.11
Peripheral vascular disease	1237		1.51 (0.66 to 3.45)	0.33
Cerebrovascular event	1237		1.22 (0.56 to 2.63)	0.62
Myocardial infarction	1237		1.32 (0.81 to 2.15)	0.26
PCI	1237		1.29 (0.75 to 2.21)	0.35
CABG	1237		2.02 (1.15 to 3.57)	**0.015**
Atrial fibrillation	1237		2.29 (1.31 to 3.98)	**0.003**
Heart failure	1237		3.98 (1.92 to 8.24)	** < 0.001**
COPD	1237		2.01 (0.97 to 4.14)	0.060
Diabetes mellitus	1237		1.58 (1.02 to 2.45)	**0.040**
Family history of CAD	1237		0.60 (0.36 to 1.01)	0.055
eGFR	1237		0.98 (0.97 to 0.99)	** < 0.001**
Frailty score	1237		1.50 (1.36 to 1.65)	** < 0.001**
PCI to multiple vessels	1237		0.84 (0.42 to 1.66)	0.61
Bifurcation disease	1237		1.27 (0.68 to 2.39)	0.45
Average Vessel Diameter	1231		0.91 (0.64 to 1.27)	0.57
Vessel Diameter ≥ 3 mm	1237		1.10 (0.65 to 1.83)	0.73

*HR* hazard ratio, *CI* confidence interval, *PCI* percutaneous coronary intervention, *CABG* coronary artery bypass grafting, *COPD* chronic obstructive pulmonary disease, *eGFR* estimated glomerular filtration rate, *DES* drug-eluting stent

Bold indicate significant result

**Table 4:** Results of multivariable Cox regression analysis for all-cause mortality

All-cause mortality (multivariate)	*N*	HR (95% CI)	*p* value
Age	1237	1.07 (1.05 to 1.10)	** < 0.001**
Hypercholesterolaemia	1237	0.59 (0.35 to 1.02)	0.057
Coronary artery bypass graft	1237	1.46 (0.82 to 2.58)	0.20
Atrial fibrillation	1237	1.24 (0.69 to 2.24)	0.47
Heart failure	1237	1.71 (0.77 to 3.80)	0.19
Diabetes mellitus	1237	1.35 (0.86 to 2.12)	0.19
eGFR	1237	1.00 (0.98 to 1.01)	0.38
Frailty score	1237	1.34 (1.21 to 1.49)	** < 0.001**

*eGFR* estimated glomerular filtration rate

Bold indicate significant result

**Fig. 4:**
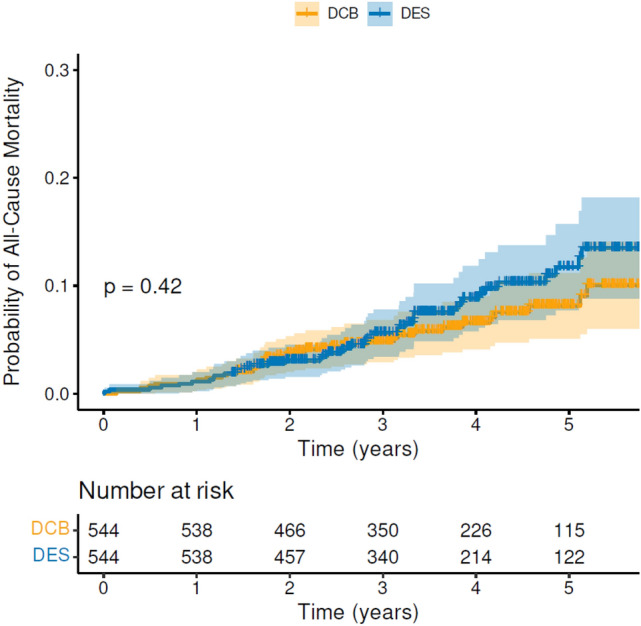
Cumulative hazard plot of all-cause mortality in propensity score-matched cohort, for DCB vs 2nd-generation DES with numbers at risk shown at the bottom of the graph. *DCB* drug-coated balloon, *DES* drug-eluting stent

Following propensity score matching 544 patients treated with DCB were matched to 544 patients treated with 2nd-generation DES. Supplementary Table 2 shows the baseline characteristics of the propensity score-matched cohort. There was no difference in all-cause mortality (Fig. 4) or any of the secondary endpoints (cardiovascular mortality, ACS, stroke/TIA, major bleeding or unplanned TLR) (Supplementary Fig. 2). Analysis of patients with treated vessel ≥ 3 mm showed that the results were unchanged (Supplementary Tables 3 and 4). In patients with large vessel treated, on multivariable Cox regression analysis, only increasing age and frailty score were significant predictors of all-cause mortality.

## DISCUSSION

Drug-coated balloon-only angioplasty is recommended in evidence-based guidelines for the treatment of in-stent restenosis and new indications are proposed in the recent International DCB Consensus Group recommendations [4, 15]. The recent BASKET-SMALL2 trial has demonstrated safety and efficacy of DCB in small vessels up to 3 years follow-up and opened up indications for DCB-only angioplasty in de novo coronary artery disease [6]. Over the last few years, registry data have demonstrated the safety of DCB-only angioplasty in de novo coronary disease [9, 10, 16]. However, the majority of these studies are limited by either long recruitment time or small numbers of patients treated with DCB-only compared to DES. In addition, very few studies directly compare DCB with DES for stable angina in de novo large-vessel disease. Therefore, it is still uncertain if DCB-only angioplasty could be part of routine clinical practice and compete safely with DES in the real world.

Our study has demonstrated that DCB-only angioplasty is safe in patients with stable angina and de novo coronary artery disease as part of a routine, clinical practice. In our institution, over the last 5 years a comparable number of patients with first presentation of stable angina due to de novo coronary disease were treated with DCB-only strategy and DES-only strategy, while at the same time, the number of patients treated with both DCB and DES remained low. There was no evidence of increased all-cause mortality with DCB-only strategy compared with DES-only approach, after > 3.5 years follow-up (median). Furthermore, there was no evidence of a difference in any of the secondary endpoints (cardiovascular mortality, ACS, stroke/TIA, major bleeding or unplanned TLR). Our results are consistent with previous registry data that have demonstrated the safety of DCB-only angioplasty and our previous study, SPARTAN DCB, which specifically showed no evidence of increased long-term mortality with DCB [10, 16]. In addition, we have demonstrated that the DCB-only strategy can compete with the DES-only strategy safely in routine clinical practice for overall mortality and all major cardiovascular endpoints, including unplanned TLR.

We included large numbers of patients with stable angina due to de novo disease and no restriction in vessel size. Approximately 73% of patients in the DCB group and 86% in the DES group had at least one vessel ≥ 3 mm treated, indicating that the great majority of patients had large vessels treated. When considering only patients with large-vessel disease, the results were similar to those observed in the whole population, showing no difference in all-cause mortality between DCB and DES (Supplementary Tables 3 and 4). These results are consistent with previous studies that have demonstrated the safety of DCB-only angioplasty for de novo disease in large vessels [17]. A large proportion (49%) of the lesions treated with DCB had residual coronary dissections, mainly grade B. Consistent with previous work from our group, the rate of acute vessel closure was very low, as only one patient had acute vessel closure within 24 h [18].

## Limitations

It is possible that the retrospective, non-randomised nature of our work from a single-centre could introduce referral bias. However, our institution is a large tertiary referral centre that provides cardiac intervention to a population over one million and has the highest implantation of DCBs for coronary artery disease in the UK [19]. Furthermore, we tried to ameliorate referral bias by including all consecutive patients fulfilling our criteria. Given that DCB-only angioplasty has a learning curve, as with most interventional techniques, our results might not be generalisable to smaller institutions with less experience in DCB-only angioplasty. In addition, it is vital to mention that even though our study is retrospective and non-randomised, our clinical database was completed prospectively, and the two groups were well balanced regarding patient characteristics. There were few differences only in terms of angiographic characteristics and recommended DAPT. Unfortunately, we do not have inflation pressures for the DCB or DES.

## Conclusion

In conclusion, this is the first study to demonstrate that DCB-only angioplasty for stable angina due to de novo disease and predominantly large vessels, is safe compared to 2nd-generation DES as part of routine clinical practice. We have demonstrated that routine DCB-only strategy in patients with stable angina due to de novo disease of all vessel sizes has no increased all-cause mortality or any other major cardiovascular endpoints, including unplanned TLR, compared to DES.

## Data Availability

Data can be available following appropriate request to the authors.
